# Autocorrelation analysis reveals widespread spatial biases in microarray experiments

**DOI:** 10.1186/1471-2164-8-164

**Published:** 2007-06-12

**Authors:** Amnon Koren, Itay Tirosh, Naama Barkai

**Affiliations:** 1 Department of Molecular Genetics, Weizmann Institute of Science, Rehovot 76100, Israel; 2 Department of Physics of Complex Systems, Weizmann Institute of Science, Rehovot 76100, Israel

## Abstract

**Background:**

DNA microarrays provide the ability to interrogate multiple genes in a single experiment and have revolutionized genomic research. However, the microarray technology suffers from various forms of biases and relatively low reproducibility. A particular source of false data has been described, in which non-random placement of gene probes on the microarray surface is associated with spurious correlations between genes.

**Results:**

In order to assess the prevalence of this effect and better understand its origins, we applied an autocorrelation analysis of the relationship between chromosomal position and expression level to a database of over 2000 individual yeast microarray experiments. We show that at least 60% of these experiments exhibit spurious chromosomal position-dependent gene correlations, which nonetheless appear in a stochastic manner within each experimental dataset. Using computer simulations, we show that large spatial biases caused in the microarray hybridization step and independently of printing procedures can exclusively account for the observed spurious correlations, in contrast to previous suggestions. Our data suggest that such biases may generate more than 15% false data per experiment. Importantly, spatial biases are expected to occur regardless of microarray design and over a wide range of microarray platforms, organisms and experimental procedures.

**Conclusions:**

Spatial biases comprise a major source of noise in microarray studies; revision of routine experimental practices and normalizations to account for these biases may significantly and comprehensively improve the quality of new as well as existing DNA microarray data.

## Background

With the availability of complete genome sequences, the ability to probe multiple genes in a single experiment using DNA microarrays provides an unprecedented tool for genomic research. Accordingly, tens of thousands of microarray experiments have been conducted to monitor changes in gene expression, identify genome-wide protein binding sites, characterize genetic variability and more. Overall, the microarray technology is of ever-increasing usefulness for multiple sorts of biological inquiries.

DNA microarrays are composed of numerous probes that usually interrogate a complete genome. The different sequence-specific probes are arrayed on a single surface either by *in-situ *oligonucleotide synthesis, or by spotting gene-specific nucleic acid fragments organized in source plates. In the latter case, robotic printers containing several print-tips are used, which partition the microarray into discrete subarray blocks representing the different tips. Subsequently, one or two labeled nucleic acid samples are hybridized to the microarray under optimally calibrated conditions and the slide is then scanned to quantify probe-specific intensity calls. The raw data obtained is usually subjected to several steps of quality control and normalization in order to remove possible biases originating in any of above steps ([1-4]).

The reliability of microarray results has been questioned due to inconsistencies in the reported data and in conclusions reached within and between different studies [5-13]. Other studies claim for adequate microarray data reproducibility [14-18]. Recently, the MicroArray Quality Control (MAQC) consortium addressed the reliability of data obtained using microarrays, by directly comparing performance across multiple platforms, test sites and replicates [19]. Concordance of qualitative gene detection calls were around 80–95% for intrasite replicates, 70–85% for intersite replicates, and 60–80% for different platforms. Alternative technologies for quantitative gene expression, such as RT-PCR, seem to provide more reliable results [19,20]. In addition, many microarray studies do not match the MAQC platform quality, experimentation expertise and relative high signal-to-noise ratios of the samples compared, and would thus generate data of yet poorer reliability. The specific technical sources underlying the suboptimal quality of the microarray technology are unclear; their identification could have a significant impact on genomic research.

Here, we investigated a specific technical effect previously reported to influence microarray data. In certain microarrays, gene probes are printed on the microarray surface according to their chromosomal position or a simple transformation thereof. When coupled to spatial biases, i.e. uneven intensity measurements across the microarray surface, such non-random probe placement designs give rise to spurious correlations between genes at particular relative positions in the genome [21-23]. This was suggested as a possible factor in the reported co-expression of adjacent genes in yeast, originally discovered in a study of gene expression during the cell cycle [24,25]. It has been suggested that print-tip effects comprise a dominant source of spatial bias underling spurious periodicities in this case [22]. Consistently, common normalization practices correct for print-tip effects ([4]) or ignore spatial biases altogether. Another study showed that inadequate cleaning of print-tips causes "carry-over" during the printing process and contributes to the generation of spurious correlations between adjacent probes [23]. However, print-tip-related effects are irrelevant to *in-situ *printed microarrays, which nonetheless exhibit spurious chromosomal-position-dependent correlations. This indicates that additional or different sources of bias are responsible for spurious correlations observed in gene expression studies.

In order to assess the extent of the effect causing spurious correlations in yeast microarray studies, we applied an autocorrelation analysis on a database of over 2000 individual microarray experiments. Remarkably, we find that spurious periodicities dominate yeast microarray datasets. Moreover, we demonstrate that they result from large and continuous spatial biases on the microarray surface, which are generated at the microarray hybridization step. The extent of such spatial biases, which are probably ubiquitous in microarray studies, has not been previously appreciated. We also show that autocorrelation can be used for the identification of aneuploidies in the strains used for expression studies, and that in certain cases, conclusions regarding segmental genetic changes can also arise spuriously.

## Results and discussion

### Autocorrelation analysis reveals spurious periodicities dependent on microarray design

**Figure 1:**
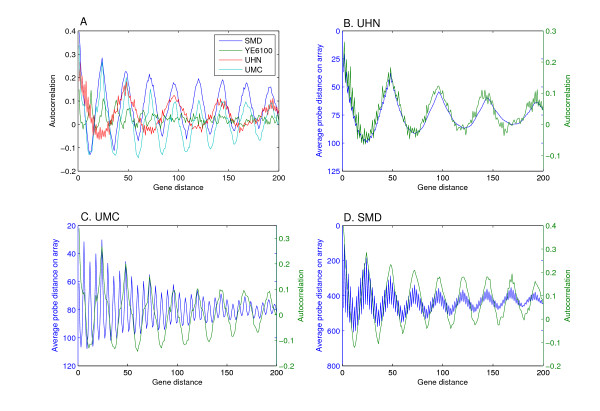
**Periodic autocorrelation dependent on microarray design**. (A) Comparison of the periodic autocorrelation in 4 related experiments. The SMD mciroarray is from Spellman *et al., *1998 [27], and the Affymetrix YE6100 is from Cho *et al., *1998 [24]. Labeled cDNA prepared form the same RNA source of cells traversing the cell cycle was hybridized onto UHN and UMC microarrays. Despite the similar, or identical, underlying biology, the autocorrelation periods are different and reminiscent of each microarray design. Thus, the characteristic period of each microarray directly corresponds to the distances of the probes on the microarray as a function of the distance of the genes in the genome. The latter is shown for the UHN (B), UMC (C), and SMD (D) microarrays alongside the autocorrelation levels for the different gene distances. Probe placement data for the Affymetrix YE6100 microarray was unavailable. In the UMC microarray, the autocorrelation period is 1/4 of the probe placement period. This is due to the precise nature of the spatial bias associated with the specific microarray analyzed (see text for details). Complete correspondence between the two parameters could be observed in other experiments (not shown).

Spurious correlations between adjacent and periodically spaced genes were previously identified by gene-gene correlations across experimental datasets encompassing multiple microarrays [21-23]. This method, however, does not discriminate between individual microarray experiments with or without associated biases. To overcome this, we used the alternative approach of autocorrelation analysis [26], in which correlations are determined between the complete gene set and matching sets shifted by gradually increasing distances along the genome. Subsequently, the autocorrelation coefficients as a function of the distances for which they were determined (Figure 1) serve to identify recurrent relations between expression levels and genomic position. Importantly, autocorrelation analysis is applied to different gene sets within individual experiments, rather than to the same genes across multiple experiments.

We tested the utility of the autocorrelation analysis on two cell cycle experiments [24,27] reported to exhibit the microarray design-related effect [21,22]. As expected, we observed very strong autocorrelation signals (Figure 1A). While autocorrelation values for gene distances of up to ~5 were highest, secondary peaks in the autocorrelation profile were also very clear. Consistent with previous observations [21,22], the secondary peaks appeared with periodicities of 24 and 13 genes for the two different experiments.

**Figure 2:**
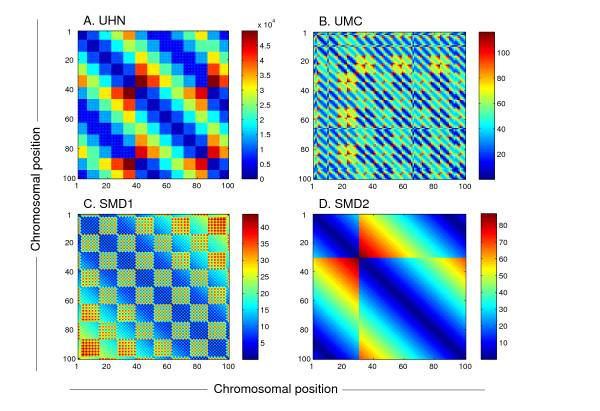
**Gene probe distance on the microarray surface as a function of the genes' chromosomal distance**. Shown is a distance matrix of the probes for the first 100 genes of chromosome 4 for each microarray design. Proximal and periodically-spaced genes are printed in proximity in each of the microarray designs shown. (A) the UHN design. (B) the UMC design. (C-D) the designs used in Spellman *et al., *1998 [27] for the α-factor (C; SMD1; same as that used in figure 1) and *cln3 *(D; SMD2) arrest and release experiments. Distances are shown in probes except for the UHN design in which they are shown in pixels because in this design there are large spaces between the different subarray blocks, making the presentation in probes less clear. The pattern in the SMD2 design is a result of a single transformation of the exact order of the genes on the chromosome region shown.

To directly demonstrate that the different periodicities represent microarray designs rather than a true biological signal, we hybridized a single RNA sample, taken from cells traversing the cell cycle, to two microarrays of different design. Indeed, the two hybridization experiments yielded different periodicities, of 24 and 48 genes respectively. Furthermore, comparison of the autocorrelation patterns with the average gene probe distance on the microarrays as a function of the genes' distance on the chromosome revealed that the different autocorrelation periodicities could be attributed entirely to the different microarray designs (Figure 1B–D). Thus, the position-related correlations in gene expression are dependent on the microarray design rather than on the underlying biology. The non-random placement of gene probes on the microarrays is visually presented in Figure 2 as the relation between chromosomal position and the corresponding distances between the probes. Genes which are adjacent on the chromosome or separated by a certain distance, characteristic of each design, are also printed in proximity on the microarray. Microarray probe placement design is manifested in the data obtained in microarray studies, and would especially be critical in studies addressing segmental genetic events or the relationship between gene position and expression.

### Presence of periodic autocorrelation patterns in multiple experiment sets

**Figure 3:**
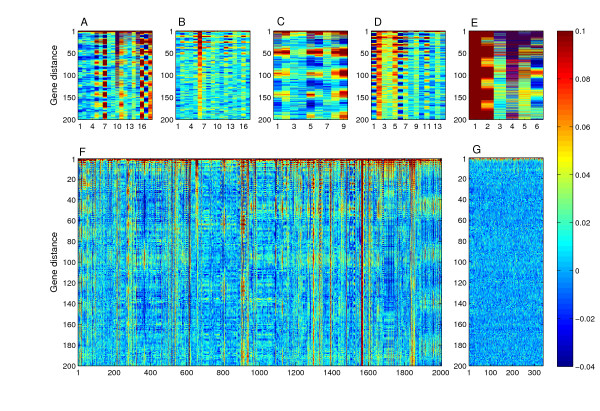
**Widespread autocorrelation patterns in microarray studies**. Autocorrelation analysis of individual microarrays from the Spellman *et al., *1998 [27] α-factor (A), Cho *et al., *1998 [24] (B; performed on Affymetrix microarrays), our unpublished cell cycle data (C), Hardwick *et al., *1999 [44] (D), and Posas *et al., *2000 [45] (E) datasets. The autocorrelation values are represented by a colorcode instead of a curve as in figure 1, and each individual experiment is represented by a seperate column in the plots. Periodic autocorrelations can be observed in most individual microarray experiments in these datasets. However, the variability of both the magnitude and the actual periods within a given dataset indicates that this effect occurs in a stochastic, rather than systematic manner. Note that in most microarray designs, both a two-gene period as well as at least one additional characteristic period could be observed in different or even the same experiment. (F) Autocorrelation analysis of 2005 yeast microarray experiments. Significant autocorrelation periodicities are manifested by values that are visually different from zero, showing that most experiments exhibit periodic autocorrelations. (G) A set of 340 experiments from a single microarray printing source are completely devoid of autocorrelation signals.

We next took advantage of the fact that autocorrelation analysis is applied to individual experiments in order to analyze in a more discrete manner three cell cycle datasets (as described above), as well as two additional datasets obtained with yet different microarray platforms. In each dataset, most experiments exhibited periodic autocorrelation, albeit with different magnitudes (Figure 3A–E). Moreover, the periods themselves varied between individual experiments within any specific dataset. Thus, a stochastic element influences the observed periodicities, affecting each particular experiment differently in both quality and quantity. Analysis of any complete dataset by gene-gene correlations would fail to show this and instead reveal only the averaged effect.

The fact that autocorrelation periodicities appear in diverse datasets prompted us to assess their extent over a wide range of microarray studies. We assembled a set of 2005 yeast microarray experiments from different laboratories, platforms and experimental procedures (see materials and methods). Numerous periodic autocorrelation patterns were observed in these experiments (Figure 3F). We quantified the extent of these periodicities by performing a second iteration of autocorrelation, which greatly enhances any periodic signals while having a minor effect on other signals, and by defining a strict significance criterion of over 20 signal points with autocorrelation r values greater than 0.05 at gene distances of up to 200. We found that 1194 of 2005 (59.5%) experiments passed this significance criterion, which is associated with a P value smaller than 10^-16^. We consider this percentage a lower bound for the fraction of experiments suffering from periodic autocorrelations. Only one source of microarrays, those produced by Rosetta Inpharmatics, did not display such periodicities, presumably due to a random probe placement design (Figure 3G). None of the 340 microarrays from this set passed our significance test for periodic autocorrelation. We conclude that the cause of spurious autocorrelations observed in the cell cycle studies dominates yeast microarray studies, and that this bias influences the final data to an extent that it can be observed as significant autocorrelation periodicities. Such spurious correlations are not confined to yeast microarrays, as they were also reported to occur in *C. elegan*s and human microarray experiments [23].

### Widespread spatial biases in microarray experiments

**Figure 4:**
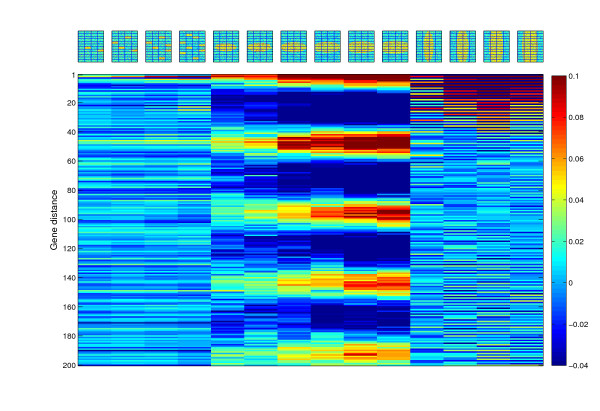
**Simulations of spatial biases**. The upper panels represent microarray images with introduced biases in yellow (arbitrary scale colorcode). The biases in panels 1–4 are subarray biases, in 5–10 horizontally-shaped biases and in 11–14 vertically-shaped biases. The lower panel shows the resulting autocorrelation pattern of each particular bias. Individual subarray effects cannot explain the observed autocorrelation periodicities, while large spatial biases give rise to various periodicities, depending on their shape.

To identify the technical source underlying the spurious autocorrelations we observed, we simulated different forms of biases introduced onto random gene expression data in the UHN yeast microarray design. We first considered biases in separate subarray blocks, which simulates print-tip-dependent biases. Such biases have previously been suggested to underlie the spurious chromosomal-position-dependent correlations [22], and they are generally regarded as an important potential source of bias in spotted microarrays. However, only weak autocorrelation signals with no periodic peaks were observed when up to eight dispersed subarray blocks deviated from the rest of the array (Figure 4). In contrast, circular shaped spatial biases larger than a subarray size order were associated with periodic autocorrelations. Furthermore, the size and shape of the spatial bias determined both the autocorrelation amplitude and the period itself. Thus, while horizontally-shaped biases resulted in a 48-gene period, a two-gene autocorrelation period was obtained in the UHN microarray design as a result of vertically-shaped biases. All the above conclusions can also be independently reached from direct examination of the probe placement information (not shown).

**Figure 5:**
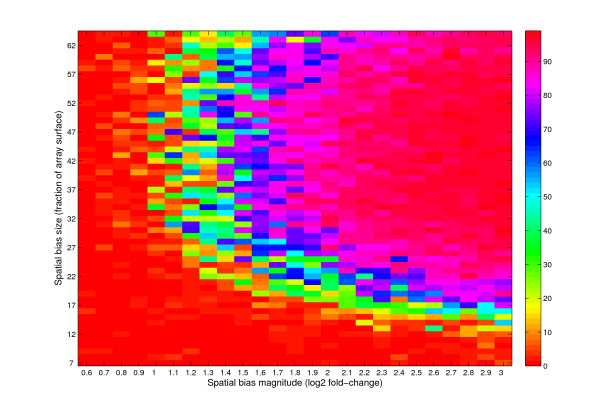
**Quantification of the dependence of autocorrelation signals on the size and magnitude of horizontally-shaped biases**. Horizontally-shaped biases (similar to those in Figure 4 lanes 5-10) of varying sizes and magnitudes of bias were introduced and the resulting autocorrelation quantified. The colorscale denotes the number of autocorrelation data points with correlation r values >0.05 in the first 200 gene distance runs. Using the same threshold as that used for evaluation of the extent of periodic autocorrelation in the real data (Figure 3F and see text), i.e. >20 signals points complying to the above criterion, it can be concluded that biases that cover more than 15% of the microarray surface and that contain at least a two-fold ratio signal are responsible for the autocorrelations observed in the real data.

To determine the significance of the spatial biases in actual studies and the degree to which they may affect the data obtained, we considered a continuum of sizes and intensity magnitudes of horizontally-shaped biases in the UHN microarray design. As can be seen in Figure 5, spatial biases that cover more than 15% of the microarray surface and that correspond to ratio measurements that deviate from the mean of the rest of the microarray by at least two-fold are responsible for generating autocorrelation patterns similar to those observed in ~60% of real experiments. As two-fold changes in expression levels have usually been regarded as a cutoff for the assignment of genes as differentially expressed, the strength of these spatial biases has a significant influence on the data. In addition, their relatively large size indicates that a significant fraction of the data is influenced.

**Figure 6:**
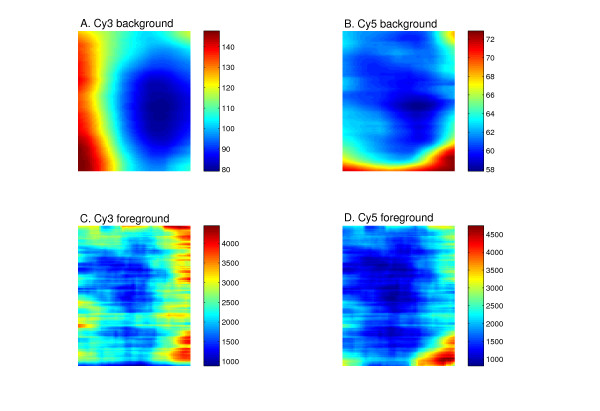
**Spatial biases differ between the foreground and background**. Images of Cy3 background (A), Cy5 background (B), Cy3 foreground (C) and Cy5 foreground (D) signal calls for a representative microarray. Shown are signals smoothed by an averaging filter to expose spatial trends. Spatial biases differ between the background and foreground as well as between the different dyes. The corresponding autocorrelation signal of the microarray shown can be seen in Figure 3C, lane 5.

In order to determine the relative contribution of foreground and background signals to spatial biases, we visually inspected several representative microarrays. As can be seen in Figure 6, spatial hybridization patterns differ between the foreground and background signals, as well as between the different dyes, accounting for the biases in the final ratio data. Moreover, the levels of the background signals are approximately 10–50-fold lower than the foreground. Thus, the autocorrelation pattern we observe in the data stems from the foreground signals. Consistently, omission of the background subtraction step did not affect the autocorrelation patterns, and neither could we prevent the appearance of autocorrelation periodicities by application of more sophisticated background subtraction methods (data not shown).

The argument that large, print-tip independent spatial biases are the cause of spurious periodicities is also consistent with the stochastic, rather than systematic nature of the appearance of periodicities (Figure 3). It is also supported by the presence of spurious periodicities in Affymetrix microarray experiments (Figure 3B), which do not contain subarrays and in which no source plates or printing tips are used. These attributes are consistent with random hybridization inhomogeneities serving as the source of spatial biases. We note that the SMD yeast microarray design is composed of only four subarray blocks, which complicated the distinction between a subarray effect and other biases and led to the previous attribution of print-tip effects to spurious correlations [22]. Accordingly, print-tip normalization seems inappropriate for correction of spatial biases, and may instead introduce unwanted edge-effects. An additional contributing factor to spatial biases was suggested to be a "carry-over" caused by inappropriate cleaning of print-tips between probe printings [23]. However, this bias produces only 0.1% noise for fully-hybridized probe spots and is at most a negligible factor relative to large spatial biases. Our simulations, which were performed on a background of random data, demonstrate that large and continuous spatial biases could solely explain all of the observed spurious periodicities.

The occurrence of large spatial biases in microarray experiments from both yeast and other organisms was previously reported ([1,28-30]). However, the use of autocorrelation analysis on data obtained from microarrays printed in a non-random manner with respect to chromosomal position has enabled us to quantify the extent of such biases over multiple experiments. We accordingly demonstrate that spatial biases occur in a majority of microarray experiments. The prevalence of such biases is probably even higher than estimated by our autocorrelation analyses, which do not detect weak or small-sized spatial biases. Importantly, the same extent of spatial biases could be expected to occur regardless of microarray design, although autocorrelation would not be useful for their identification in such cases. Since we analyzed experiments from a variety of platforms, laboratories and procedures, we infer that spatial biases are a ubiquitous characteristic of microarray studies in general.

The above conclusions emphasize the need to apply a spatial bias correction step when analyzing microarray data. We tested several methods for spatial bias correction and found that virtually any method, including print-tip normalization and corrections of spatial gradients, effectively eliminate all periodic autocorrelation signals (data not shown). However, none of these capture the actual nature of the spatial trends and can introduce additional biases and edge effects. Instead, a method termed MANOR (Micro-Array NORmalization) has previously been presented [29], which accounts for both local, abrupt spatial signal changes, as well as continuous intensity gradients. MANOR combines a spatial segmentation procedure with a two-dimensional Loess regression and is optimized to preserve the true biological signal when correcting for spatial biases. It is publicly implemented in an R package (available at [31]). We consider MANOR the most suitable algorithm for the correction of spatial biases in microarray experiments in general. Although originally implemented in spatial normalization of array-CGH data, our demonstration of widespread spatial biases in various sorts of microarray experimental procedures makes it relevant also to non-CGH experiments, for which it comparably removes autocorrelation periodicities (not shown).

### Identification of aneuploidy by autocorrelation

**Figure 7:**
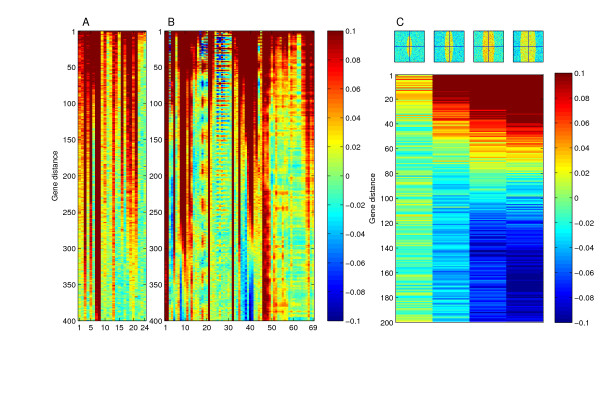
**Identification of aneuploidy by autocorrelation**. (A) Gene expression comparisons of strains with verified differences in chromosome copy number result in continuous stretches of high autocorrelation values, due to the similarity in expression measurements over long genomic intervals. Data shown is from strains that contain aneuploidies (lanes 1-22) or segmental duplication of 56 or 28 genes (lanes 23-24, respectively) [32, 33]. (B) CGH experiments are frequently associated with similar autocorrelation patterns. The datasets are those described in Dunham *et al.*, 2002 [46], and Dunn *et al.*, 2005 [47]. Note that periodic autocorrelations are also observed in several of these experiments. (C) In specific microarray designs, such as the SMD design, spatial biases of certain shapes can generate "spurious aneuploidies". Note that the autocorrelation is shown for different gene distances than in (A-B).

In addition to periodic autocorrelations, we observed many experiments with long tracts of continuously high autocorrelation (Figure 3F; a particular example can be seen in Figure 3E lane 1), indicative of segments of multiple genes with similar data measurement levels. We suspected that aneuploidies or segmental copy number variations in the cells used for the experiments may be the reason for the presence of these patterns in the data. Consistently, all previously confirmed aneuploid strains used in a study of gene expression in deletion mutants [32,33] exhibited continuously strong autocorrelation tracts (Figure 7A). Strains with segmental duplications of 58 and 28 genes were also clearly identified by high autocorrelation tracts, proportional in gene distance to the length of the genetic alteration (Figure 7A lanes 24–25). A similar pattern was observed in many comparative genome hybridization (CGH) experiments (Figure 7B), presumably representing the genetic alterations in the studied strains.

Several of the long autocorrelation tracts we observed in our expression data assembly may represent additional cases of aneuploidies in the strains used for generating the data. However, others occur in experiments in which the control and experiment samples were taken from genetically-identical culture samples. We suspected that the autocorrelation patterns observed in these cases may be the result of another ramification of the effect of specific spatial biases coupled to microarray design. Indeed, we found that narrow and long spatial biases in the SMD microarray design can cause such an aneuploidy-like signal, which is nonetheless spurious in origin (Figure 7C). Thus, spatial biases can lead to false identification of genetic alterations in studies based on non-random microarray designs.

## Conclusions

In this study we have demonstrated the utility of autocorrelation analysis for the efficient identification and filtering of spurious chromosomal-position-dependent correlations. In particular, we provide compelling evidence for the prevalence of large spatial biases in microarray studies, to an extent unappreciated thus far. Our conclusions are based on data simulations, the stochastic nature of spurious autocorrelation patterns, and the existence of spurious correlations in spotted as well as Affymetrix microarray experiments. Although we have identified spatial biases by their manifestation in the form of periodic autocorrelation, which in itself depends on microarray design, their frequency of occurrence should be constant over many microarray platforms irrespective of design. Our simulations suggest that spatial biases are commonly associated with signal changes of a factor of two or more over large portions of the data, which represents a significant extent of bias and a potent source of false data. Spatial biases can accordingly explain the many cases of poor or suboptimal reproducibility in microarray studies. We suggest that normalization methods that correct for spatial biases, such as MANOR [29], should be routinely applied when analyzing microarray data. Re-analysis of existing data should also consider such spatial biases and their effect on the data. Finally, future improvement of microarray data quality should concentrate on overcoming spatial biases, mainly by optimization of hybridization procedures.

## Methods

### Experimental procedures

BY4743 diploid *Saccharomyces cerevisiae *cells grown in YPD media were arrested in late G2 by addition of 10 μg/ml Nocodazole (Sigma) for 1.5 hours and subsequently released into the cell cycle. Sample preparation, microarray hybridization and data extraction were performed as previously described [34]. The data was background subtracted and not normalized for print-tip-dependent or other spatial biases. Microarrays used were the UHN Y6.4k4 PCR-product microarrays representing complete yeast ORFs (University Health Network, Toronto), and the UMC Utrecht *S. cerevisiae *16K array version 1.1, which consist of 70-mer oligonucleotide probes unique for each yeast gene. The raw data and log2-transformed ratio data ordered by genomic position, for each of the microarray designs, can be found at our website, at [35].

### External datasets used

All public data analyzed is background-subtracted intensity or ratio calls without any spatial bias normalization.

Yeast cell cycle expression data corresponds to the α-factor arrest and release experiment from Spellman *et al.*, 1998 [27], hybridized onto SMD *Saccharomyces cerevisiae *Array y744, and the *cdc15 *temperature-sensitive mutant arrest and release experiments from Cho *et al., *1998 [24], hybridized onto Affymetrix YE6100 microarrays. For Figure 1, we used the 42 and 110 minute time points from these studies, respectively.

We analyzed a total of 2438 separate microarray experiments from the following sources: 1) A previously described yeast gene expression database ([36], details of which can be found at [37]), which was assembled in 2002 and includes experiments performed on a variety of microarray platforms, including 125 experiments from early versions (YE6100 and S98) of Affymetrix yeast expression microarrays. 2) The complete database of yeast microarray studies from the Stanford Microarray Database (SMD; [38,39]) excluding experiments already included in the former database and those with >1000 missing values. This database also covers experiments recently performed. 3) A set of 113 ChIP-on-chip experiments [40]. We used the P value data for this experimental set since it constitutes the relevant user-level data for these experiments; the ratio data from which the P value data was derived yielded the same results in terms of autocorrelations. These experiments add to another 83 ChIP-on-chip experiments from the SMD database.

Experiments performed with deletion strains harboring verified aneuploidies [32], as well as the *ymr031w-a *deletion strain, and comparative genome hybridization (CGH) experiments from the SMD database gave a unique autocorrelation signature and were analyzed separately. An additional 340 experiments from three studies performed on microarrays designed by Rosetta Inpharmatics [33,41,42] showed no autocorrelation patterns, presumably due to random probe placement (we could not verify this), and were thus separated from the rest of the database and treated as a negative control.

### Autocorrelation analysis

The log2-transformed ratio data (or intensity in Affymetrix experiments) was used for autocorrelation analyses. Genes were ordered according to their genomic position, taken from the *Saccharomyces *Genome Database (SGD; [43]). Pearson correlation coefficients were determined for distances of between one gene and the size of the gene list-1, according to the formula: Autocorr(X, i) = Corr(X(1:L-i), X(i:L)), where X is the ordered data, i is the gene distance, and L is the length of the gene list. Missing values in the data were given a log2 ratio value of zero; this caused a decrease in the autocorrelations values to some extent, but retained the actual periods themselves.

In order to evaluate the significance of periodic patterns in the autocorrelations, we performed an autocorrelation analysis on the autocorrelation data itself. Any periodic signals are significantly enhanced by this procedure, while having only a marginal effect on non-periodic signals. We chose a significance criterion of second-iteration autocorrelation r values of >0.05, and demanded that at least 20 data points out of the first 200 pass this criterion in order for an experiment to be regarded as containing significant periodicities. These figures were chosen since they yielded zero false positives in the control dataset (Figure 3G) and, by visual inspection, identified the maximal number of true periodicities in the studied dataset. The P value of this criterion is <10^-16 ^(using the binomial distribution on randomized autocorrelation data, which distributed approximately normally with mean ~0 and standard deviation ~0.01).

### Simulations of spatial biases

Random expression data was generated by permutating measurements from a given experiment. Either individual subarray blocks, or circular spatial shapes, were given a ten-fold higher value in one channel. Circular shapes were defined as complying to the formula: ((X−C1)⋅F)2+(Y−C2)2≤R
 MathType@MTEF@5@5@+=feaafiart1ev1aaatCvAUfKttLearuWrP9MDH5MBPbIqV92AaeXatLxBI9gBaebbnrfifHhDYfgasaacH8akY=wiFfYdH8Gipec8Eeeu0xXdbba9frFj0=OqFfea0dXdd9vqai=hGuQ8kuc9pgc9s8qqaq=dirpe0xb9q8qiLsFr0=vr0=vr0dc8meaabaqaciaacaGaaeqabaqabeGadaaakeaadaGcaaqaaiabcIcaOiabcIcaOiabdIfayjabgkHiTiabdoeadnaaBaaaleaacqaIXaqmaeqaaOGaeiykaKIaeyyXICTaemOrayKaeiykaKYaaWbaaSqabeaacqaIYaGmaaGccqGHRaWkcqGGOaakcqWGzbqwcqGHsislcqWGdbWqdaWgaaWcbaGaeGOmaidabeaakiabcMcaPmaaCaaaleqabaGaeGOmaidaaaqabaGccqGHKjYOcqWGsbGuaaa@4401@, where X and Y are the coordinates of the spots that fall within the bias shape, C_1 _and C_2 _represent the center coordinates (set at the center of the microarray surface), F is a circularity factor (set at 1 for horizontally-shaped circular biases and for generating Figure 5, and at 8 for vertically-shaped biases), and R is the radius of the bias. Subsequently, autocorrelations were calculated on the log2-transformed ratio data for each introduced bias.

## Authors' contributions

AK conceived of the study, designed the study, performed the experiments and the computational analyses and drafted the manuscript. IT participated in the computational analyses and helped to draft the manuscript. NB designed the study and helped to draft the manuscript. All authors read and approved the final manuscript.
